# Readmission and mortality during the first year after an acute stroke: A prospective cohort study from Cameroon

**DOI:** 10.1371/journal.pone.0311893

**Published:** 2024-10-28

**Authors:** Clovis Nkoke, Ahmadou Musa Jingi, Jean Jacques Noubiap, Cyrille Nkouonlack, Debimeh Njume, Anastase Dzudie

**Affiliations:** 1 Buea Regional Hospital, Buea, Cameroon; 2 Clinical Research Education, Networking and Consultancy (CRENC), Douala, Cameroon; 3 Faculty of Health Sciences, University of Bamenda, Bamenda, Cameroon; 4 Center for Heart Rhythm Disorders, University of Adelaide, Adelaide, SA, Australia; 5 Faculty of Health Sciences, University of Buea, Buea, Cameroon; 6 Faculty of Medicine and Biomedical Sciences, University of Yaounde 1, Yaounde, Cameroon; PLoS ONE, UNITED STATES OF AMERICA

## Abstract

**Background:**

Hospital readmission after discharge for stroke is associated with high morbidity and mortality. There is a paucity of data on the burden of stroke readmission in most sub-Saharan African countries. We aimed to determine the rate, reasons and predictors of hospital readmission and the mortality rate within 12 months of discharge among stroke survivors in Cameroon.

**Methods:**

This prospective cohort included patients who survived hospitalization for an acute stroke and who were discharged from two referral hospitals in the capital city of Cameroon between January 2013 and December 2013.We performed logistic regression analysis to identify demographic and clinical factors associated with readmission within 1 year of discharge and causes of readmission.

**Results:**

Of the 254 consecutive patients admitted for acute stroke, 198 were discharged alive. There were 107 (54%) males, and their mean age (SD) was 61.7 (13.9) years (range: 10 to 95 years). A total of 30 (15.2%) patients were readmitted following discharge from the index admission over a mean follow-up time of 286.9 (127.6) days. Of these, 14 (46.7%) were discharged and 16 (53.3%) died after readmission. Thirty percent (30%) of readmissions occurred within the first month. The most frequent causes of readmissions were infections (30%) and recurrent stroke (26.7%). Factors associated with readmission in bivariate analysis were initial admission temperature > 37.5°C (risk ratio [RR]: 1.3, p = 0.021) and initial admission Glasgow Coma Score < 14 (RR: p = 1.23, p = 0.019). After stratified adjustment for age and sex, temperature > 37.5°C (adjusted RR: 1.3, 95% CI: 1–1.7, p = 0.036) and GCS <14 (adjusted RR: 1.23, 95% CI: 1–1.6, p = 0.041) were associated with readmission.

**Conclusion:**

Readmission after discharge for stroke was common with nearly one third occurring within the first month and more than half dying following readmission. The most common causes of readmission were infections and recurrent stroke.

## Introduction

Stroke is a major public health problem globally. It is associated with considerable morbidity and mortality with those in low-and middle-income countries especially sub-Saharan Africa being disproportionately affected [1, 2]. Stroke is a major cause of hospitalization in sub-Saharan Africa and the incidence is rising as a result of epidemiological transition [3, 4].

Readmission after hospitalization for stroke is frequent. All-cause readmission has been reported in 13%–62% of patients within one year after index stroke [5–10]. Stroke readmission is associated with increased morbidity and mortality [11]. Although there has been significant improvement in the acute management and secondary prevention of stroke in high income countries, there has been limited progress in the management of stroke in Sub-Saharan Africa [12]. A significant proportion of readmission is probably due to unaddressed medical issues at the time of discharge, non-optimal post-hospital management, and rehabilitation of victims [13].

While the stroke incidence and fatality rates are substantial in Cameroon, there is scarcity of data regarding readmission and long-term mortality in stroke survivors in the country [4, 14]. Hence, we conducted this study to determine the one-year readmission and mortality rates among patients discharged alive after admission for acute stroke in Cameroon, and factors associated with poor outcomes.

## Methods

### Study design and setting

We carried out a prospective cohort study in two referral hospitals in Yaoundé, the capital city of Cameroon. These tertiary centers serve as teaching hospitals and have a catchment population of over 2 million inhabitants. The study methodology has been published previously [14]. The study was conducted in the medical units of the Yaounde Central Hospital and the Yaounde General Hospital. These were the two largest hospitals in the Capital City of Cameroon (Yaounde), with a catchment population of about two million individuals in 2012 at the time the study was conducted. These are public hospitals located in the heart of the city with easy road access both from within and most neighboring rural areas and, however patient transport to these medical facilities are in general through private cars renting system (Taxi), personal cars with limited emergency medical system and limited access to ambulance. There was no universal health coverage in Cameroon, however patient presenting with acute medical conditions are taken care of regardless of their health coverage, stabilized and will be required later to cover the cost related to care. Stroke patients were admitted for the most part in medical units and no acute stroke therapy is available to date in the country. The medical units were the largest departments of each of the hospitals. The two units had a cumulative capacity of 141 beds distributed across seven subspecialty units including neurology. The department of Medicine at the Yaounde Central Hospital is staffed with 29 specialist physicians and that of the Yaounde General hospital by 15 specialists, and in both hospitals working in collaboration with emergency physicians, intensivists, neuroradiologists, neurosurgeons, general practitioners, and junior specialist physicians in training. Admission protocols were similar in both hospitals. Patients admitted to the units were referred from the emergency departments, other departments, out-patient clinics and other hospitals.

### Study participants

All consecutive patients with a diagnosis of stroke were seen for possible inclusion in the study. Verbal consent was obtained from patients or a surrogate and the telephone numbers of those who accepted to be included were obtained as approved by the ethics committee. Data collected was part of routine clinical care in this cohort. We applied the WHO definition of stroke as “rapidly developing clinical signs of focal (or global) disturbance of cerebral function, with symptoms lasting 24 h or longer or leading to death, with no apparent cause other than of vascular origin [14].

### Variables

The interview was used to obtain participants’ age, sex, marital status, profession, level of education, residence. Information relating to stroke onset, pre-existing risk factors including hypertension, diabetes mellitus, dyslipidemia, atrial fibrillation, previous stroke, heart failure. Hypertension, diabetes mellitus, smoking, ischemic heart disease (with/without ECG changes), alcohol consumption, and heart failure history were based on history from patients or surrogates. This information was supplemented from data obtained from hospital records. Dyslipidemia and atrial fibrillation were based on a blood test and ECG done during admission.

The initial neurological assessment was performed within 24 hours of admission. Stroke severity was assessed using the National Institute of Health stroke scale (NIHSS) on admission [15]. Routine laboratory and imaging workup served as the basis for stroke etiology and risk factors investigation.

Stroke outcome was assessed starting in the hospital for in-hospital complications and discharge status. Patients surviving index hospitalization were systematically assessed at 1 month, 3 months, 6 months, 9 months, and 12 months post-stroke during hospital post-stroke follow up appointment visit or by phone interview.

### Definition of variables

Readmission within 1 year of the index hospital discharge was used as the outcome variable. Independent variables examined for association included age, sex, marital status, history of vascular risk factors (diabetes mellitus, hypertension, and cigarette smoking), history of stroke, alcohol consumption, length of hospital stay, and discharge disposition. Discharge disposition was categorized as a discharge to home or rehabilitation center. These were variables identified in publications examining hospital readmission within one year [5–10]. We carried out this work in accordance with the declarations of Helsinki.

### Statistical analysis

The data were analyzed using SPSS version 26. For the univariate analyses, we present continuous variables as means (SD) and discrete variables as frequencies and percentages. In bivariate analyses, we estimated the risk (Risk Ratio) of readmission for selected variables identified in previous studies. We used the stratification method to assess for independent predictors of readmission (adjusting for age and sex). We used the Kaplan-Meier survival curves to estimate the readmission-free rates at 1, 3, 6, 9, and 12 months excluding those who died without readmission or lost to follow-up. The statistical significance was set at p-value <0.05.

## Results

### General characteristics

**Fig 1:**
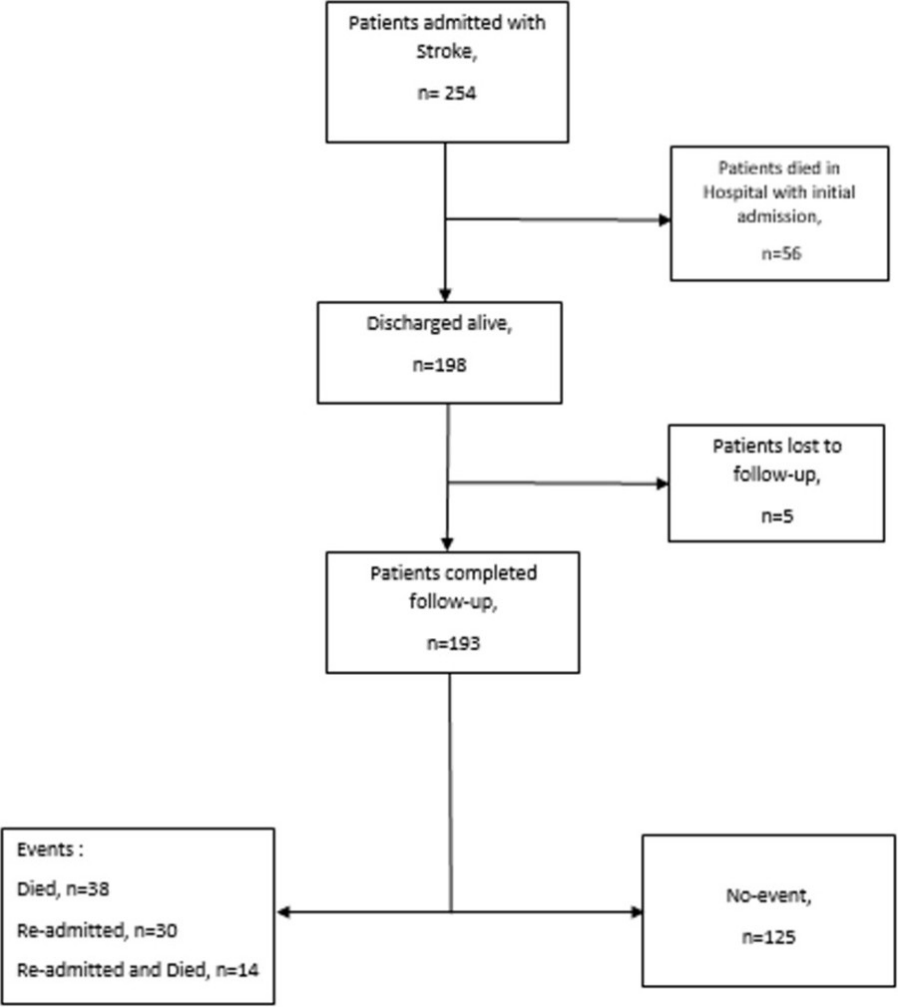
Flow chart of participants.

During the study period, a total of 254 patients were hospitalized for acute stroke. Of these, 198 were discharged alive after a mean (SD) hospital stay of 12.6 (7.8) days and were followed-up for 12 months. Five patients were lost to follow-up (Fig 1).

The discharge orientation was home in 179 (89.9%), rehabilitation centre in 19 (9.6%), and unidentified destinations in 1 (0.5%) patient. The mean age (SD) of the study population was 61.7 (SD 13.9) years (range: 10 to 95 years), and 107 (54%) were males. Hypertension was the most frequent risk factor (64.1%) and a previous stroke was reported by 27 (13.6%) of patients. Ischemic stroke was the most frequent stroke type (66.7%) (Table 1).

**Table 1:** General characteristics of the participants.

Variables	Overall, n(198)	Re-adm, n(30)	No Re-adm, n(168)	p-value
Age, mean (SD)	61.7 (13.9)	63.8 (14.1)	61.3 (13.8)	0.369
Male sex, n (%)	107 (54)	15 (50)	76 (45.2)	0.630
Length of Hospitalization, mean (SD)	12.6 (7.8)	14.5 (6.9)	12.3 (8)	0.164
**History**				
Hypertension, n (%)	127 (64.1)	16 (53.3)	111 (66.1)	0.180
Diabetes, n (%)	37 (18.7)	6 (20)	31 (18.5)	0.841
Previous Stroke, n (%)	27 (13.6)	6 (20)	21 (12.5)	0.270
Heart Failure, n (%)	9 (4.6)	0 (0)	9 (5.4)	0.194
Atrial fibrillation, n (%)	7 (3.5)	1 (3.3)	6 (3.6)	0.948
Alcohol, n (%)	54 (27.3)	8 (26.7)	46 (27.4)	0.936
Ever smoked, n (%)	34 (17.2)	5 (16.7)	29 (17.3)	0.648
**Clinical presentation and Discharge**				
Admission NIHSS, mean (SD)	8.96 (5.2)	9.7 (5.5)	8.8 (5.1)	0.378
Discharge NIHSS, mean (SD)	7.69 (4.9)	8.2 (5.1)	7.6 (4.9)	0.513
Admission mRS, mean (SD)	3.67 (1.03)	3.9 (1)	3.6 (1)	0.125
Discharge mRS, mean (SD)	3.56 (1.4)	3.7 (1.4)	3.5 (1.4)	0.644
SBP, mean (SD) mmHg	177 (34.7)	181.7 (46)	176.2 (12.8)	0.426
DBP, mean (SD) mmHg	102.4 (21.9)	108 (28.6)	101.4 (20.4)	0.132
Heart rate, mean (SD)	79.6 (12.9)	80.8 (13.8)	79.4 (12.8)	0.573
Temperature, mean (SD)	37 (0.6)	37.2 (0.7)	37 (0.5)	0.176
Glasgow score, mean (SD)	14.4 (1.4)	13.7 (2)	14.5 (1.3)	**0.005**
Discharged to rehabilitation, n (%)	19 (9.6)	5 (16.7)	14 (8.3)	**0.020**
**CT Stroke sub-type**				
Ischemic, n (%)	132 (66.7)	19 (63.3)	113 (67.3)	0.134
Hemorrhagic, n (%)	52 (26.3)	11 (36.7)	41 (24.4)	
Undetermined, n (%)	14 (7.1)	0 (0)	14 (8.3)	

The most frequent causes of readmissions were recurrent stroke (26.7%) and infections (30%) (Table 2). Factors associated with readmission in bivariate analysis were initial admission temperature > 37.5°C (RR: 1.3, p = 0.021) and admission Glasgow Coma Score (GCS) < 14 (Table 3). Hospital stay > 12 days, discharge NIHSS score > 7, and discharge to rehabilitation were not associated with readmission. After stratified adjustment for age and sex, temperature > 37.5°C (adjusted RR [aRR]: 1.3, 95% CI: 1–1.7, p = 0.036) and GCS <14 (aRR: 1.23, 95% CI: 1–1.6, p = 0.041) were risk factors for readmission.

**Table 2:** Causes of readmission.

Variable	Frequency (n)	Percentage (%)
Hypertensive crises	2	6.7
Recurrent stroke	8	26.7
Fatigue	2	6.7
Severe anemia	1	3.3
Seizures	2	6.7
Bedsores	1	3.3
Refuse to eat	1	3.3
GIT bleeding	1	3.3
Alcohol encephalopathy	1	3.3
Infections*	9	30
Undefined	2	2
Total	30	100

*Infections: urinary tract: 1; meningitis: 1; sepsis: 1; severe malaria: 1; diabetic foot: 1; pneumonia: 3; undefined: 1

**Table 3:** Risk factors of readmission.

Variable	Re-admission, n (%)	Unadjusted risk	
		RR (95% CI)	*p*-value
Sex			
Female	15(16.5)	1.03 (0.9–1.2)	0.629
Male	15(14)	1	
Age (years)			
>60	16 (15.4)	1.01 (0.9–1.1)	0.923
≤60	14 (14.9)	1	
Hospital Stay (days)			
>12	14 (20.3)	1.1 (0.96–1.3)	**0.140**
≤12	16 (12.4)	1	
Previous stroke			
Yes	6 (22.2)	1.1 (0.9–1.4)	0.270
No	24 (14)	1	
Discharge NIHSS > 7			
Yes	18 (18.4)	1.08 (0.96–1.2)	**0.212**
No	12 (12)	1	
DischargemRS> 3			
Yes	20 (15.5)	1.01 (0.9–1.1)	0.850
No	10 (14.5)	1	
Temperature> 37.5°c			
Yes	7 (31.8)	1.3 (1–1.7)	**0.021**
No	23 (13.1)	1	
GCS < 14			
Yes	9 (29)	1.23 (1–1.6)	**0.019**
No	21 (12.6)	1	
Ischemic Stroke			
Yes	19 (14.4)	0.97 (0.9–1.1)	0.674
No	11 (16.7)	1	
Discharge rehabilitation			
Yes	5 (26.3)	1.17 (0.9–1.5)	**0.153**
No	25 (14)	1	

### Stroke readmission rates, determinants and causes

**Fig 2:**
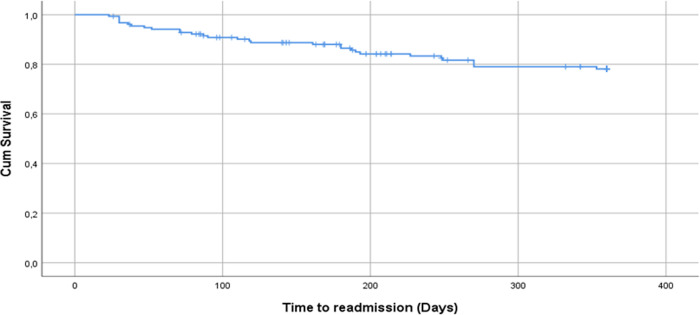
Kaplan-Meier survival plot (event free) post stroke discharge.

A total of 30 (15.2% of total discharge) patients were readmitted over a mean follow-up time of 286.9 (127.6) days (95% CI: 269.1–304.8). Of these, 14 (46.7%) were discharged and 16 (53.3%) died during their hospital stay after readmission (Fig 1). Nine (30%) readmissions occurred within 1 month, 7 (23.3%) by Month 3, 9 (30%) by month 6, 5 (16.7%) by month 9, and no readmission by Month 12. Using the Kaplan-Meier analysis (excluding dose who died without readmission or were lost to follow-up), the readmission free at Months 1, 3, 6, 9, and 12 were 96.8%, 90.8%, 88%, 79.1%, and 78.1% respectively (Fig 2).

## Discussion

The objective of this study was to determine the prevalence of stroke readmission, its determinants and causes of readmission in a cohort of stroke survivors discharged after an index hospitalization for stroke in the capital city of Cameroon during a one-year follow up. Our results show that 15.2% of stroke survivors were readmitted and 53.3% died during readmission. The most common causes of readmission were infections and recurrent stroke.

To the best of our knowledge, this is the first study reporting on stroke readmission in a prospective follow up Cameroon. The rate of readmission in our study was 15.2%. This falls within the range that has been reported in other studies with readmission rates of 13% -62% [5–10]. But most of these reports were from high income countries where the acute stroke management, post stroke care and rehabilitation are substantially different from that in low income countries. Thus, direct comparisons are difficult. However, these differences can be due to differences in methodologies and differences in stroke care.

Data on the predictors of readmission after stroke are inconsistent. The identified factors significantly associated with readmission in our study were temperature >37.5°C and Glasgow coma score<14. Glasgow coma score is an index of stroke severity and predictor of poor outcome [16]. Thus, patients with severe stroke were more likely to be readmitted. The NIHSS which is another measure of stroke severity was not an independent factor associated with readmission in our study, a finding that was consistent with prior publications [6]. The other predictor of readmission was fever. This finding could also reflect the fact that stroke patients are at increased risk of infections that will present with fever. However, fever after stroke can also be due to immune system activation or effects of the brain lesion on thermoregulatory centers, and is sometimes referred to as “central fever” [17]. Central fever can be difficult to distinguish from fever due to infections [17]. Regardless of the causes, fever after stroke is associated with poor prognosis [18]. Some factors independently associated with readmission in prior studies were hypertension, atrial fibrillation; male sex, age, previous and post-stroke functional status length of hospital stay [6, 19, 20]. Stroke type was not associated with readmission, which was in agreement with previous studies [19, 21].

In our study, the most common causes of readmission were infections followed by a recurrent stroke. This is consistent with findings from other studies [20]. Infections after stroke are common, and the prevalence has been reported to be as high as 30% [22]. These infections are associated with higher morbidity and mortality [23]. This highlights the need for surveillance for infections in patients discharged for stroke. Previous reports have shown that in stroke survivors, cardiac disease, recurrent stroke, infection and falls are common causes for readmission, with recurrent stroke being particularly common in the first months [6–9, 24, 25].

Our findings demonstrated that more than half (53.3%) of those readmitted died during the subsequent hospitalization. Previous reports have shown that stroke readmission is associated with poor outcome [6]. Infections were one of the most common causes of readmission in our study. Post-stroke infections whether occurring early or late are associated with an increased risk of death [26]. A previous study from Cameroon showed that stroke survivors had a 43% higher risk of dying from a recurrent stroke compared to those with first ever stroke against a background of unfavorable risk factor profile [27]. This highlights the need to intensify secondary prevention strategies in stroke survivors.

### Limitations

Our study is limited by the small sample size which does not permit us to identify all potential predictors of readmission. We might have underestimated readmission as some patients might have been admitted to other hospitals other the two hospitals where the study was conducted. Also, some patients might have died at home or before reaching the hospital. Despite this limitation, our study is the first to report on stroke readmission in Cameroon.

## Conclusion

Readmission after discharge for stroke was common with nearly one third occurring within the first month and more than half dying following readmission. The most common causes of readmission were infections and recurrent stroke.

## Supporting information

S1 Data (XLSX)
